# Uncovering Nanoindention Behavior of Amorphous/Crystalline High-Entropy-Alloy Composites

**DOI:** 10.3390/ma17153689

**Published:** 2024-07-25

**Authors:** Yuan Chen, Siwei Ren, Xiubo Liu, Jing Peng, Peter K. Liaw

**Affiliations:** 1 Hunan Province Key Laboratory of Materials Surface/Interface Science Technology, Central South University of Forestry Technology, Changsha 410004, China; 20221100238@csuft.edu.cn; 2 College of Mechanical and Vehicle Engineering, Hunan University, Changsha 410082, China; rsw1428@hnu.edu.cn; 3 Department of Materials Science and Engineering, The University of Tennessee, Knoxville, TN 37996, USA; pliaw@utk.edu

**Keywords:** amorphous/crystalline high-entropy alloy, nanoindentation, mechanical properties, microstructure

## Abstract

Amorphous/crystalline high-entropy-alloy (HEA) composites show great promise as structural materials due to their exceptional mechanical properties. However, there is still a lack of understanding of the dynamic nanoindentation response of HEA composites at the atomic scale. Here, the mechanical behavior of amorphous/crystalline HEA composites under nanoindentation is investigated through a large-scale molecular dynamics simulation and a dislocation-based strength model, in terms of the indentation force, microstructural evolution, stress distribution, shear strain distribution, and surface topography. The results show that the uneven distribution of elements within the crystal leads to a strong heterogeneity of the surface tension during elastic deformation. The severe mismatch of the amorphous/crystalline interface combined with the rapid accumulation of elastic deformation energy causes a significant number of dislocation-based plastic deformation behaviors. The presence of surrounding dislocations inhibits the free slip of dislocations below the indenter, while the amorphous layer prevents the movement or disappearance of dislocations towards the substrate. A thin amorphous layer leads to great indentation force, and causes inconsistent stacking and movement patterns of surface atoms, resulting in local bulges and depressions at the macroscopic level. The increasing thickness of the amorphous layer hinders the extension of shear bands towards the lower part of the substrate. These findings shed light on the mechanical properties of amorphous/crystalline HEA composites and offer insights for the design of high-performance materials.

## 1. Introduction

High-entropy alloys (HEA) have received significant attention due to their remarkable properties, including high strength, excellent thermal stability, and wear resistance [1,2,3,4]. In comparison to traditional alloys, CoCrFeNi system HEAs are relatively lightweight and demonstrate higher yield strength and superior specific strength [3,5]. As a result, they have great potential for application in key industrial sectors such as aerospace, vehicle manufacturing, armor protection, and marine industries. In practical applications, HEA devices are inevitably exposed to various mechanical loads. The plastic deformation and associated damage progression in HEA materials play a crucial role in determining the reliability of HEA devices in service, making further investigation essential.

Several studies show that the trade-off between strength and ductility is addressed by the unique morphology of alloys [6,7]. Recently, there has been a growing interest in amorphous/crystalline (A/C) composites, as they combine the unique features of both amorphous and crystalline phases, and enhance the mechanical properties of materials through additional strengthening and toughening mechanisms, which can improve the application prospects of alloys in a wide range of fields. Initially, the A/C composite was introduced to tackle the strength–ductility trade-off of Mg alloys at room temperature [8,9,10,11]. The A/C Mg alloy demonstrates its ability to enhance the plasticity of Mg alloys through an unconventional plastic deformation mechanism [8]. In a subsequent experimental study, a nanosized A/C Mg alloy achieved near-theoretical strength and good plasticity [9]. A significant discovery shows that the elemental diffusion from the amorphous phase to the A/C interface can enhance the amorphization of the crystalline phase in A/C Mg alloys [10]. Following these successes with Mg alloys, A/C Mg alloys have been applied to enhance the mechanical properties of HEAs [12,13,14]. An A/C HEA composite prepared by magnetron sputtering technology showed hardness of 8.9 GPa, surpassing that of the most well-known CoCrFeMnNi HEAs [12]. In a separate study, an experimentally fabricated A/C HEA composite exhibited a yield strength close to theoretical levels and a uniform plastic strain exceeding 45% under compression [13]. Furthermore, the impact of the position and thickness of the amorphous layer on the deformation behavior and mechanical properties of A/C HEA composites was studied through molecular dynamics (MD) simulation [14]. Despite the progress made in researching A/C HEA composites, this field is still emerging, with numerous phenomena waiting to be fully understood and explored.

The interaction behavior between dislocations and amorphous phases plays a crucial role in determining the mechanical properties of A/C composite. However, understanding this interaction mechanism during the deformation process of HEA composites through experiment remains challenging. In such cases, MD simulation has proven to be a valuable alternative for revealing the microscopic deformation mechanism of materials, as it can analyze microstructural interactions at the atomic scale. By employing MD simulation, researchers can gain insight into the relationship between the outstanding mechanical properties and microstructural evolution of A/C HEA composites, aiding in their design and selection [15,16,17,18,19,20]. The impact of factors such as amorphous layer spacing, indenter velocity, and indenter radius on the interaction between dislocations and amorphous phases was examined in A/C CoCrFeMnNi HEA composite through MD simulation. It was found that the critical indentation depth and force necessary for plastic deformation in HEAs decrease as the indenter velocity increases, which is opposite to the behavior seen in single-phase crystalline HEA composite. This research offers theoretical insights that can guide the design and fabrication of high-performance A/C HEA composite.

In the present work, MD simulations were employed to investigate the deformation mechanisms and mechanical properties of A/C HEA composites during nanoindentation. The thickness of the layers was systematically varied, and the mechanical behavior of the nanolaminates was studied using atomistic simulations during nanoindentation. By analyzing deformation snapshots and creating mechanism maps based on the microstructure, the interaction mechanism between dislocations and amorphous phases in A/C dual phase HEA composites was revealed, enhancing new understanding of the transformation of deformation mechanisms. The study also examined how shifts in these mechanisms impacted the mechanical properties of the composites. Overall, the current research established a theoretical framework for a better understanding of the mechanical properties of HEA composites at the nanoscale, providing theoretical guidance for the design and preparation of high-performance A/C dual phase HEA.

## 2. Simulation Method and Model

**Figure 1:**
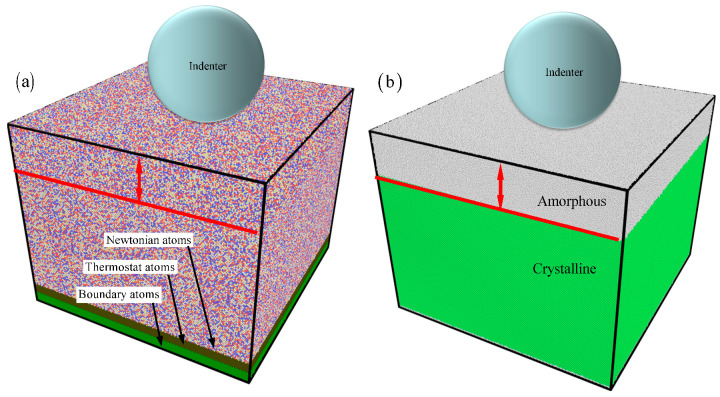
The nanoindention model of A/C HEA composites. 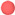 Fe, 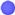 Co, 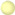 Cr, and 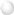 Ni. (**a**) The atoms are colored by the atom types. (**b**) The atoms are represented by different colors based on the structure types.

Figure 1 shows the nanoindention simulations of amorphous/crystalline HEA FeCrCoNi structures, showcasing a model that includes both amorphous and crystalline HEA components. The dimension of the amorphous/crystalline HEA FeCrCoNi sample is 464 Å × 455 Å × 460 Å. To explore how size impacts nanoindention deformation in amorphous/crystalline HEA composites, the amorphous HEA layer thicknesses range from 2 to 12 nm. The amorphous/crystalline HEA FeCoCrNi comprises randomly distributed Fe, Co, Cr, and Ni atoms. To obtain the amorphous HEA model, it was melted at 2500 K in an isobaric-isothermal ensemble (NPT) before quenching it from 2500 K to 300 K at a cooling rate of 10 K/ps [21,22]. According to the experiment [8], the crystallographic orientation of the crystalline HEA layer is set as [0 1 1] along the *x*-axis, $\left[1\;\bar{1}\;1\right]$ along the *y*-axis, and $\left[2\;1\;\bar{1}\right]$ along the *z*-axis. The amorphous/crystalline FeCoCrNi HEA consists of three layers (Figure 1a): the boundary layer, thermostat layer, and Newtonian layer [23,24]. (i) The boundary layer, with a thickness of 5 nm, is anchored at the bottom of the HEA sample. (ii) The thermostat layer, with a thickness of 5 nm, is maintained at a constant temperature of 300 K using the velocity scaling method to simulate heat dissipation during the indentation process. (iii) The atom movements in the Newtonian layer follow Newton’s second law. The *x*- and *y*-directions are subject to periodic boundary conditions, while the *z*-direction has free boundary conditions.

During the indentation process, an indenter with a constant speed of 10 m/s along the *z*-direction moves towards the sample (Figure 1a). This indentation speed used in previous work has been widely adopted [25,26]. While it is faster than the speed used in the experiment, atomic simulation requires significant computing resources in order to achieve the most accurate prediction results possible. The microcanonical ensemble (NVE) is employed for the stages of relaxation and nanoindentation. Following a relaxation stage, the nanoindentation process is initiated. The parameters for the MD simulation are outlined in Table 1. Here, the maximum depth ensures the existence of elastic deformation in the crystal layer, preventing unreasonable results caused by the hardening of the bottom layer. In addition, based on the trial-and-error method, other simulation parameters were selected. The embedded atom method (EAM) potential is commonly utilized in the study of solidification, microstructure evolution, and plastic deformation [27,28,29]. Using the current EAM potential, previous studies have investigated the microstructure evolution, mechanical properties, and strengthening mechanisms in the single crystal HEA [29], dual-phase HEA [6], nanocrystalline HEA [30], and amorphous HEA [31,32]. A repulsive rigid spherical indenter is employed to model the interaction between the indenter and the HEA composite [17,18,19,20,21]. The time step was 1 fs in all MD simulations, using the large-scale atomic/molecular massively parallel simulator (LAMMPS) (version LAMMPS-64bit-latest-MPI.exe) [33].

The microstructural evolution was visualized using the Ovito software (version Ovito 2.9.0.exe) [34]. The common neighbor analysis (CNA) was employed to identify the different microstructures following deformation. In this analysis, red atoms indicate the presence of stacking faults, green atoms signify the face-centered cubic (FCC) structure, and white atoms correspond to dislocation cores, interface structures, and amorphous regions. Here, the effect of vacancy on indentation behavior is neglected due to its extremely low vacancy concentration.

## 3. Result and Discussion

### 3.1. The Nanoindention Process

**Figure 2:**
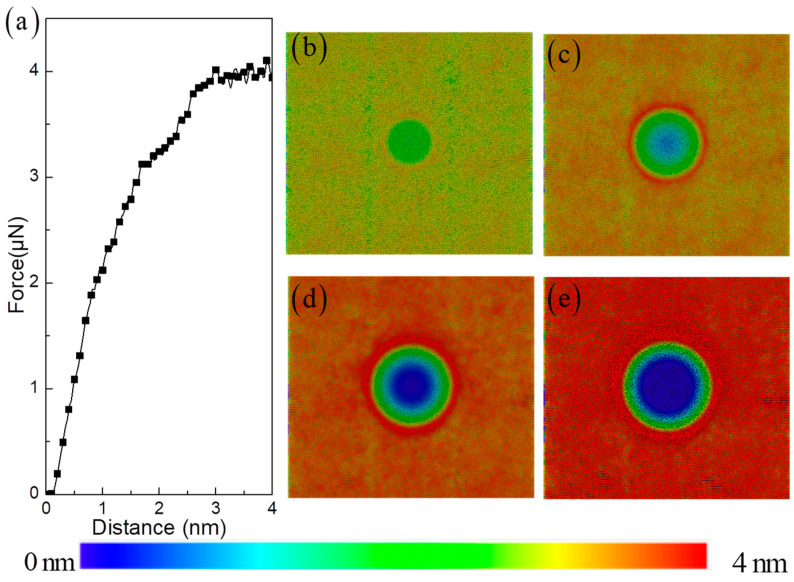
Relationship of indentation force versus indentation depth in A/C HEA composite (**a**). The surface morphology with increasing indentation depth: (**b**) 1 nm, (**c**) 2 nm, (**d**) 3 nm, and (**e**) 4 nm.

From the previous experiments [35,36], the evolution of the indenter load can provide insight into the elastic and plastic deformation of the matrix metal. Here, Figure 2a shows the relationship between indentation force and the depth of indentation. The process of indentation is divided into four distinct stages, which include elastic deformation, yielding, strain hardening, and softening. Here, the elastic deformation is defined as the process in which material undergoes deformation without the formation of dislocations. The yielding is defined as the initial nucleation stage of dislocations. The strain hardening is defined as numerous dislocation proliferations, and the softening is defined as the process in which dislocations continue to proliferate at a relatively constant indentation force. Therefore, it is essential to examine the changes in surface morphology and microstructure within each of the four time intervals, in order to gain a better understanding of the deformation behavior of HEA composites at the atomic level. Figure 2b–e illustrate the evolution process of surface morphology as the indentation depth increases. In the initial stage of elastic deformation, the uneven distribution of elements within the crystal causes heterogeneity in surface tension. This trend leads to fluctuations in the height of surface atoms in specific regions of the HEA [37].

However, once the deformation reaches the yield stage, this effect begins to diminish as plastic deformation takes over. In particular, the outermost circle of the indenter’s contact area shows a significant buildup of atoms, causing them to shift outward. The subsequent stages of hardening and constant load (softening) display distinct fluctuations in the local atomic height of the surface (Figure 2b–e). This is a result of the intense local plastic deformation altering the distribution of atoms, pulling them towards the direction of the indenter in a network-like fashion [17,18,19,20]. This phenomenon differs slightly from observations at a macroscopic level, where deformation is primarily concentrated within 1.2 times the indenter diameter range. At the microscale, this region can extend beyond twice that size, highlighting the importance of atomic size effects in adapting to large local deformations.

**Figure 3:**
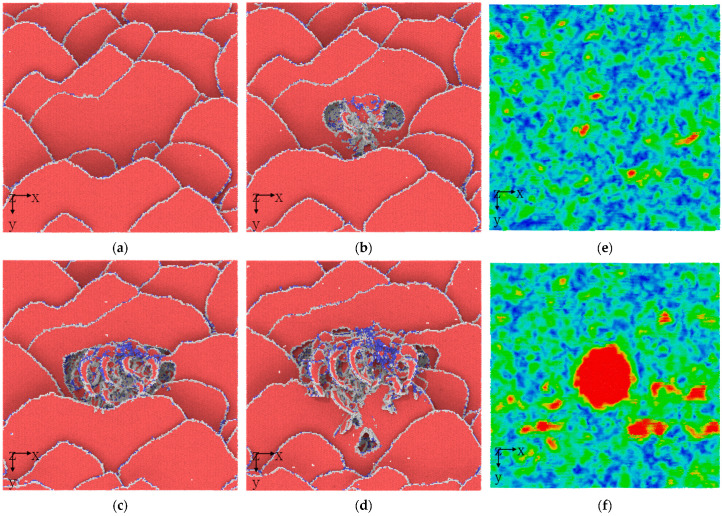
The instantaneous microstructure with the increasing indentation depth: (**a**) 1 nm, (**b**) 2 nm, (**c**) 3 nm, and (**d**) 4 nm. (**a**) The microstructure near the interface at the elastic deformation, (**b**) initial dislocation generation, (**c**) dislocation multiplication, and (**d**) extension of dislocation slip. 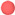 FCC structure, 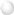 dislocation core structure, and 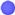 BCC structure. The shear strain distribution of the interface at the indentation depth: (**e**) 1 nm, and (**f**) 2 nm.

Figure 3a–d shows the microstructure evolution process induced by plastic deformation directly underneath the indenter. Figure 3a clearly shows that the conventional plastic deformation stage, as determined by stress–strain curve division, is not suitable for accurately describing the indentation plastic behavior of nanoscale HEA composites. This clearly reveals the generation of a large number of stacking faults. The severe mismatch at the interface between amorphous and crystalline materials, coupled with the rapid accumulation of elastic deformation energy (Figure 3e,f), has led to the observation of a large number of plastic deformation dislocation behaviors in the traditional sense. The previous in situ TEM nanoindentation result shows some primary dislocations start to nucleate and propagate in the Ti_2_AlN phase from the Ti_2_AlN//TiAl incoherent interface [38]. The dislocation pile-up with distinct cross-slip characteristics is found in the A composite TiZrHfNb_0.5_Cu_0.5_Be_0.5_, consisting of a high-entropy phase TiZrHfNb and an amorphous matrix TiZrHfCuBe [39]. In addition, some atomic simulation works further elucidate the existence of elastic deformation energy in the interface [40,41].

After entering the yield stage, plastic deformation further activates dislocations in other directions, and then reacts violently and increases in value with the stacking dislocations generated during the elastic deformation stage. This phenomenon has gone through both hardening and softening stages. The stacking fault structure continues to disappear, and its strengthening mechanism correspondingly decreases [6,17,18,19,20]. However, the corresponding number of dislocations continues to increase, and the contribution of dislocation strengthening continues to rise. Finally, at the highest point of strain hardening, the two reach their equilibrium at the highest peak. Subsequently, the strengthening of dislocations reaches its peak. This result has been widely observed in previous experiments or simulations [38,39,40,41]. In order to further understand this process, the corresponding evolution process of dislocation lines should also be presented.

**Figure 4:**
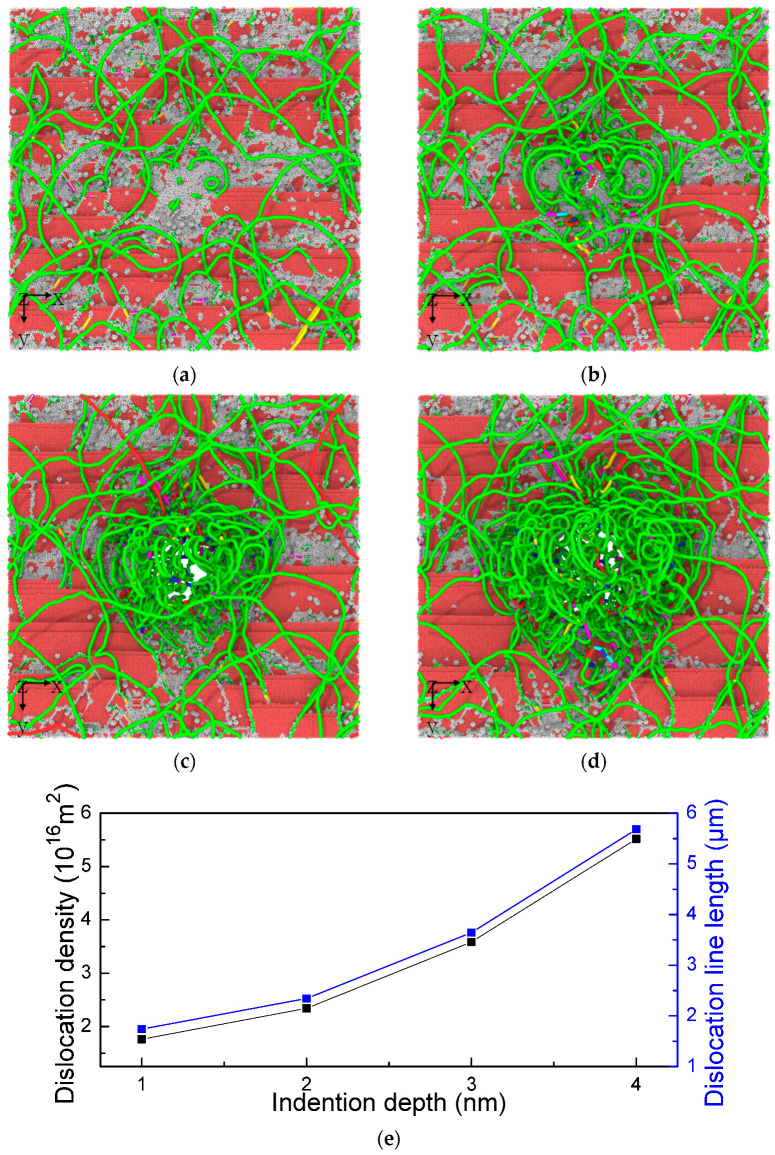
The dislocation variation at the indentation depth: (**a**) 1 nm, (**b**) 2 nm, (**c**) 3 nm, and (**d**) 4 nm. The blue line is perfect dislocation, the green line is Shockley partial dislocation, the red line is other dislocation, the sky-blue line is Frank partial dislocation, the pink line is stair-rod dislocation, and the yellow line is Hirth dislocation. (**e**) The dislocation density and dislocation line length versus indentation depth in amorphous/crystalline HEA composite.

The shape and characteristics of a large number of Schockley partial dislocations are presented in Figure 4. The continuous microstructure evolution depends on the depth of the indenter. As shown in Figure 4a, the dislocation structures of other configurations react with the prior dislocations to produce new dislocation characteristic configurations. This process shows the HEA composites are in the plastic deformation stage (Figure 4b). Subsequently, a large number of dislocation reactions accelerate the spread of dislocation characteristics in this region towards the surrounding areas. Seriously twisted and bent dislocation lines fold into a spatially curved elliptical structure (Figure 4c). This extremely different dislocation behavior from that of traditional alloys leads to a sharp increase in dislocation density directly below the indenter, which also means that the high dislocation density region is bound to form a high-stress environment (Figure 4d). This process will also produce strain gradient effects. The presence of surrounding dislocations restricts the free slip of dislocations below the indenter, while the amorphous layer restricts the possibility of dislocation movement or disappearance towards the substrate [6,17,18,19,20]. The continuous increase in dislocations, along with the disappearance of dislocations around the indenter, enters another evolutionary process, ultimately leading to an increase in Shockley partial dislocation density in the material matrix (Figure 4e).

**Figure 5:**
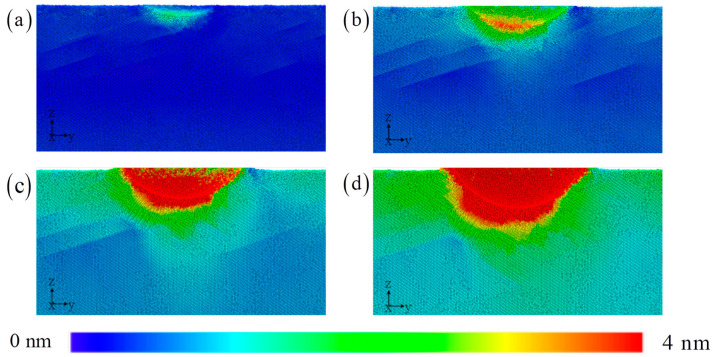
The displacement distribution with the increasing indentation depth: (**a**) 1 nm, (**b**) 2 nm, (**c**) 3 nm, and (**d**) 4 nm.

The distribution of atom displacement is used to understand how the process from elasticity to plastic deformation is formed through local atomic motion. During the process of elastic deformation, the orientation of the single crystal matrix leads to uneven atomic motion and still has directionality (Figure 5). It can be roughly observed that the atoms are moving downwards to the left in the direction of 45°, which is consistent with the direction of maximum shear stress. During the plastic deformation stage, this process is further enhanced. The HEA is divided into left and right sides based on the direction of the head movement. The atomic motion on the left is significantly higher than that on the right. Heterogeneous atomic motions also have a corresponding impact on the distribution of microstructures [6,17,18,19,20]. From this it can be inferred that the strain gradient effect on both sides is significantly different. The localized plastic flow is nonuniform. The conventional observation shows that high atomic motion displacements are still concentrated in the area around the indenter, rapidly weakening and spreading outwards.

**Figure 6:**
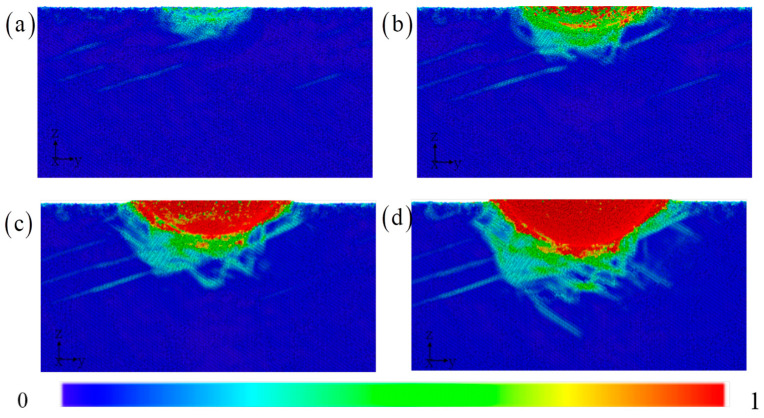
The shear strain distribution of A/C HEA with the increasing indentation depth: (**a**) 1 nm, (**b**) 2 nm, (**c**) 3 nm, and (**d**) 4 nm.

The shear strain of atoms is an effective characterization method that directly describes the plastic deformation characteristics of amorphous regions. Shear bands are defined as effective and distinct band configurations formed when the local strain exceeds 0.2, as shown in Figure 6. The shear band is generated around the indenter and moves downward along the sliding direction, gradually decaying and terminating inside the matrix [6,17,18,19,20]. This feature is different from the dislocation type and does not necessarily terminate at the surface or grain boundaries. Similar to the displacement distribution of atoms, the shear strain on the left side is related to that on the right side. Atomic motion induces the normal and shear strains.

**Figure 7:**
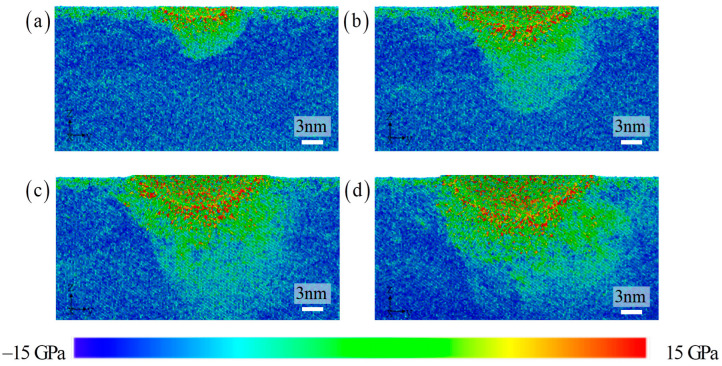
The stress distribution with the increasing indentation depth: (**a**) 1 nm, (**b**) 2 nm, (**c**) 3 nm, and (**d**) 4 nm.

Figure 7 illustrates the distribution of atomic stress. The distribution of atomic stress extends deeper towards the substrate, compared to the characteristics of atomic displacement and strain distribution. The high strain region and high stress region of HEA composites are not completely consistent. This will lead to a coupling effect on material properties due to the dual effect of the strain gradient and stress gradient [6,17,18,19,20]. The effect range of stress gradient is wider during the stage of severe plastic deformation of materials. This comparison result allows us to evaluate two differences at the atomic scale. Previous results have also shown that there is a difference between the two.

### 3.2. Effect of Amorphous Layer Thickness

**Figure 8:**
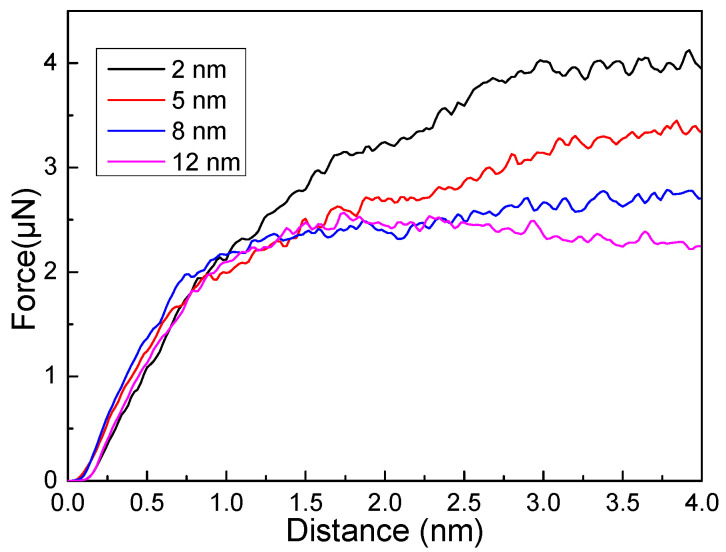
The indentation force versus indentation depth in amorphous/crystalline HEA composite with different amorphous layers of 2, 5, 8, and 12 nm.

In order to clarify the influence of amorphous layer thickness on the indentation performance of HEA composite materials, Figure 8 shows the evolution relationship between indentation force and indentation depth under different amorphous layer thicknesses. During the elastic deformation stage, the thickness of the amorphous layer has little effect on the performance. After the yield stage, it has a significant impact on the indentation performance. The specific result is that the small thickness of the amorphous layer causes the great indentation force [6,17,18,19,20]. Compared to the crystalline layers, amorphous layers have poorer deformation ability and naturally limited strain-hardening ability. The dislocation multiplication within the crystal layer provides a greater contribution to the strengthening. This may be due to an increase in the thickness of the amorphous layer, a shortening of the strain-hardening deformation stage, and an increase in the significant softening stage. The specific details are that the average indentation force gradually decays from 2790 μN to 2367 μN.

**Figure 9:**
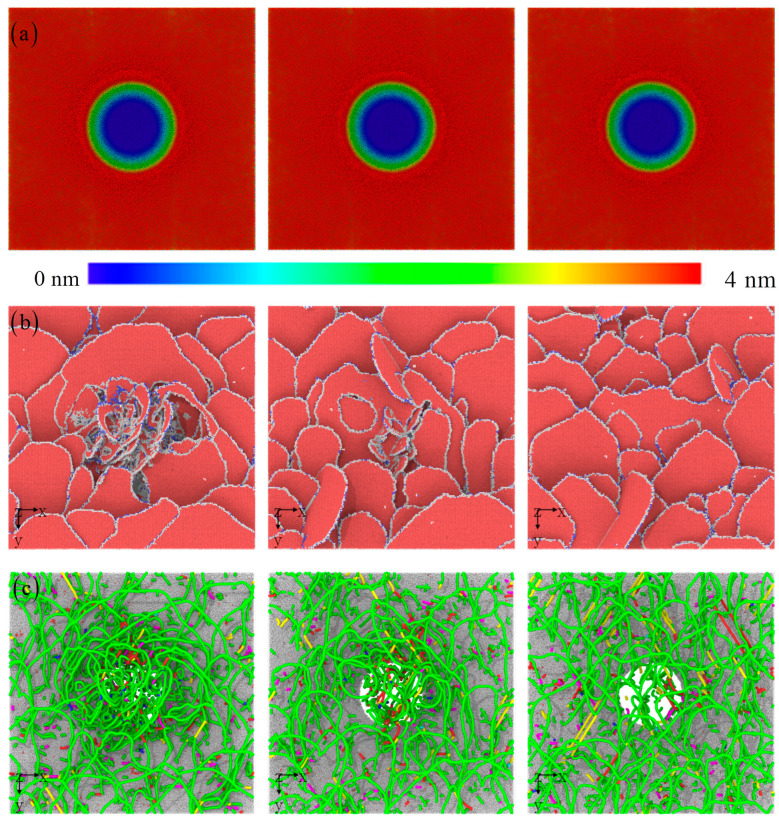
The surface morphology, instantaneous microstructure, and dislocation with the increasing amorphous layer thickness: (**a**) 5 nm, (**b**) 8 nm, and (**c**) 12 nm.

Figure 9 illustrates the evolution of morphology, microstructure, and dislocation patterns. The characteristic of atomic stacking around the indenter is similar for different thicknesses [6,20,42]. The deformation characteristics of surface atoms at other positions are also similar. This result indicates that changes in the thickness of the amorphous layer will not cause significant changes in the surface morphology of the material. Under load, there are no large areas of high shear strain inside the amorphous layer. There is a change in the relative position of local atoms due to shear strain, which leads to local lattice distortion. At the macroscopic level, it causes inconsistent stacking and movement trends of surface atoms, resulting in local bulges and depressions.

However, the evolution of the microstructure within the crystal layer is crucial for the sub-indication. The amorphous layer can effectively release stress, and higher stress–strain can only be transmitted to the crystalline layer in a certain proportion. This will inevitably lead to significant differences in microstructure evolution. Figure 9b shows that the distribution and configuration of stacking faults below the indenter are different. Specifically, directly below the indenter, the formation of new dislocations interacts with those initially formed near the interface, resulting in a change in dislocation density [6,20,42]. To clarify this process, the types of dislocation lines are presented in Figure 9c. As previously speculated, a large number of new dislocations have indeed formed below the indenter. When the thickness reaches a critical value, the amorphous layer can almost completely isolate the transfer of stress or strain to the crystalline layer. In other words, more stress can be absorbed by the amorphous layer, reducing the plastic deformation of the crystalline layer. Therefore, if the thickness of the amorphous layer exceeds a critical value, only elastic deformation occurs in the crystalline layer. More detailed discussions would remain in the future work. The critical thickness not only depends on the thickness of the amorphous material, but also on the elements of the amorphous material.

**Figure 10:**
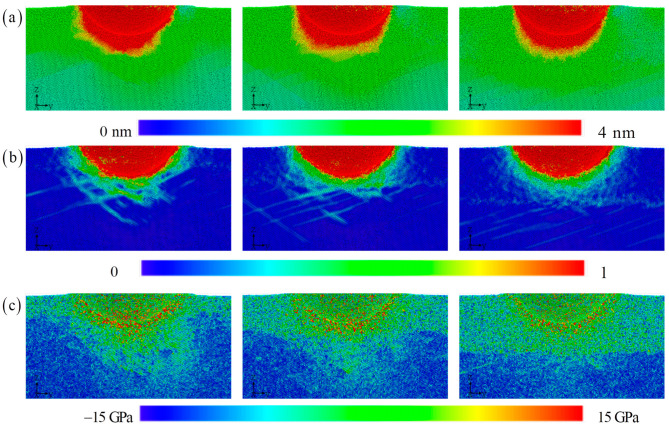
The distribution of the displacement, strain, and stress with the increasing amorphous layer thickness: (**a**) 5 nm, (**b**) 8 nm, and (**c**) 12 nm.

The displacement field, strain field, and stress field are described in Figure 10. The displacement distribution shape of the atoms below the pressure head is asymmetric to the lower left, with a flat bottom for symmetric distribution and a sharp bottom for symmetric distribution. Therefore, even if the types and positions of atoms in the upper layer of the amorphous layer are exactly the same, as the thickness of the amorphous layer increases, it will still lead to this peculiar atomic motion phenomenon. Under the load, the upper atoms are stacked in different configurations of the lower layer, and the difference in local mechanical properties of the lower layer will inevitably change the position of each atom in the upper layer [20,43]. This effect gradually decays with increasing indentation depth. The differences in local shear bands further validate this observation. The most obvious area is still the contact area around the indenter, which ultimately affects the change in indenter pressure. The increase in amorphous layer thickness suppresses the extension of shear bands towards the lower part of the substrate. Instead, it releases high strain within the matrix through local dispersion. The more direct and clear revelation of the stress field reveals the amorphous and crystalline layers. Although the current work can reveal deformation mechanisms of amorphous/crystalline HEA composites at the atomic scale, atomic simulation itself has limitations in simulating size and high deformation rates, and the predicted results have certain deviations in large-sized models. Therefore, there is an urgent need to explore deformation mechanisms based on multiscale simulation methods.

### 3.3. Strengthening Model

In order to quantitatively evaluate the strengthening effect of A/C HEA composite, the amorphous phase strengthening and typical microstructure strengthening in HEA should be considered. The previous studies prove that the interaction between crystalline and amorphous phases not only activates the dislocations motion in the crystalline region, but also activates the shear transition region, resulting in a softening effect related to the thickness of the amorphous zone [44,45]. This is a means to achieving the synergy of strength and ductility [44]. Thus, the strength influenced by the amorphous alloys can be expressed as:

(1) $$\sigma_{A}=\sqrt{{\sigma_{a0}}^{2}+\frac{\psi }{t}}$$

where $\sigma_{a0}$ is the strength of the amorphous bulk, and is obtained from the MD tension simulation of the amorphous FeCoCrNi HEA [6]. ψ is an empirical constant. *t* is the thickness of the amorphous layer.

For the HEA crystalline phase, the solid solution strengthening caused by the mismatch of atomic size and shear modulus is the notable strengthening mechanism [46,47]. Due to the fact that the elements of HEA are solutes to each other, the overall solid solution strengthening effect is attributed to the individual contributions of each element.

(2) $$\sigma_{ss}=\sum_{i=1}^{n}c_{i}\sigma_{ss}^{i}$$

where $\sigma_{ss}^{i}$ is the mismatch strengthening effect caused by the element *i*, which can be further expressed as:

(3) $$\sigma_{ss}^{i}=A\mu c_{i}^{2/3}{\delta_{i}}^{4/3}$$

where *A* is the material parameter and the value is 0.04. $\mu =\sum_{i}^{n}c_{i}\mu_{i}$ is the shear modulus obtained by the mixing rule [48]. $\delta_{i}$ is the mismatch parameter, which can be written as:

(4) $$\delta_{i}=\xi (\delta \mu_{i}^{2}+\beta^{2}\delta r_{i}^{2}{)}^{1/2}$$

where the value of ξ is related to the lattice type of the metal, and ξ=1 for FCC metals, ξ=4 for BCC metals. β is a parameter related to the type of dislocation; for screw dislocation 2<β<4, for the edge dislocation β≥16 [47]. $\delta r_{i}$ and $\delta \mu_{i}$, respectively, are the atomic size mismatch and the modulus mismatch caused by the solute atom *i.*

(5) $$\delta r_{i}=\frac{\delta r_{ijkl}^{ave}-\delta r_{jkl}^{ave}}{c_{i}}$$

(6) $$\delta \mu_{i}=\frac{\delta \mu_{ijkl}^{ave}-\delta \mu_{jkl}^{ave}}{c_{i}}$$

Here, the *ijkl* HEA is considered to be formed by introducing solute atom *i* into the *ijk* HEA. $\delta r_{ijkl}^{ave}$ and $\delta \mu_{ijkl}^{ave}$ are the average atomic size mismatch and the average shear modulus mismatch of the *ijkl* HEA, which can be calculated as:

(7) $$\delta r^{ave}=\sum_{i}^{n}\sum_{j}^{n}c_{i}c_{j}\delta r_{ij}=\left(c_{1},c_{2},\cdots ,c_{n}\right)\left(\begin{array}{cccc} \delta r_{11} & \delta r_{12} & \cdots & \delta r_{1n}\\ \delta r_{21} & \delta r_{22} & \cdots & \delta r_{2n}\\ \vdots & \cdots & \ddots & \vdots \\ \delta r_{n1} & \delta r_{n2} & \cdots & \delta r_{nn} \end{array}\right)\left(\begin{array}{c} c_{1}\\ c_{2}\\ \vdots \\ c_{n} \end{array}\right)$$

(8) $$\delta \mu^{ave}=\sum_{i}^{n}\sum_{j}^{n}c_{i}c_{j}\delta \mu_{ij}=\left(c_{1},c_{2},\cdots ,c_{n}\right)\left(\begin{array}{cccc} \delta \mu_{11} & \delta \mu_{12} & \cdots & \delta \mu_{1n}\\ \delta \mu_{21} & \delta \mu_{22} & \cdots & \delta \mu_{2n}\\ \vdots & \cdots & \ddots & \vdots \\ \delta \mu_{n1} & \delta \mu_{n2} & \cdots & \delta \mu_{nn} \end{array}\right)\left(\begin{array}{c} c_{1}\\ c_{2}\\ \vdots \\ c_{n} \end{array}\right)$$

where $\delta r_{ij}$ and $\delta \mu_{ij}$ are the atomic size mismatch and the modulus mismatch between the atom *i* and the atom *j*.

(9) $$\delta r_{ij}=2\left(r_{i}-r_{j}\right)/\left(r_{i}+r_{j}\right)$$

(10) $$\delta \mu_{ij}=2\left(\mu_{i}-\mu_{j}\right)/\left(\mu_{i}+\mu_{j}\right)$$

where $r_{i}$ and $r_{j}$ are the atom size of element *i* and *j*, respectively. $\mu_{i}$ and $\mu_{j}$ are the shear modulus of element *i* and *j*, respectively.

For the present MD simulation model, the HEA crystalline phase is considered to be a nanolayer, and the strengthening effect derived from the interface can be written as [49,50]:

(11) $$\sigma_{b}=M\frac{\mu b_{p}\sin\theta }{8\pi d}\left(\frac{4-\upsilon }{1-\upsilon }\right)\ln\left(\frac{\alpha d}{b_{p}\sin\theta }\right)$$

where *M* is the Taylor constant, *d* is the thickness of the nanocrystalline layer, $b_{p}$ is the magnitude of the Burger vector of partial dislocation, θ is the angle between the slip plane and the interface, υ is Poisson’s ratio, and α is the core cut-off parameter.

The contribution of activated dislocation in the HEA crystalline phase to strength cannot be ignored. The dislocation resistance is expressed as [50]:

(12) $$\sigma_{d}=M\xi \mu b\sqrt{\rho }$$

where ξ is the Taylor constant, *b* is the magnitude of the Burger vector of dislocation, and ρ is the dislocation density originating from MD simulation.

Thus, considering the above microstructure strengthening effect, the overall HEA crystalline phase strengthening is written as:

(13) $$\sigma_{C}=\sigma_{b}+\sigma_{ss}+\sigma_{d}$$

Considering the characteristics of A/C HEA composite, the overall strength depends on the proportion of each structure [6,44]:

(14) $$\sigma_{y}=f_{1}\sigma_{A}+f_{2}\sigma_{C}$$

where *f*_1_ and *f*_2_ are the volume fractions of the amorphous phase and crystalline phase, respectively. The physical parameters of elements are listed in Table 2, and the material parameters are listed in Table 3.

**Figure 11:**
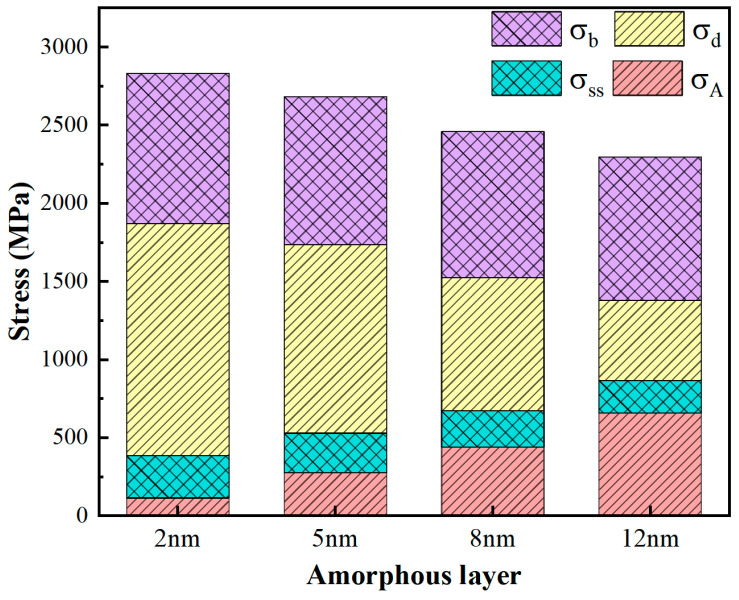
The strength of A/C HEA composite with different amorphous layers thickness of 2 nm, 5 nm, 8 nm, and 12 nm.

For the A/C HEA composite with different amorphous layer thicknesses of 2 nm, 5 nm, 8 nm, and 12 nm, the contribution of the amorphous phase strengthening, solid solution strengthening, interface strengthening, and dislocation strengthening to strength is calculated, as shown in Figure 11. The specific contribution values of each strengthening mechanism are listed in Table 4. The results indicate that when the thin amorphous layers thickness is 2 nm, at this time the volume fraction of the amorphous phase is only 4.3%, the contribution of amorphous phase strengthening is weak. The strength is determined by the microstructure in the crystalline phase, and dislocation strengthening is the dominant strengthening mechanism. As the thickness of the amorphous layer increases, the overall strength gradually decreases, which is consistent with the previous study [44]. The increase in the volume fraction of the amorphous phase leads to an increase in the proportion of its strengthening mechanism. The solid solution strengthening is an inherent characteristic of HEA, which is less affected by changes in amorphous thickness. In addition, due to the proportion of crystalline phase decreases, the deformation mechanism dominated by the dislocation movement in the crystalline phase transforms to amorphous deformation. The activated dislocation density decreases, resulting in a decrease in the dislocation strengthening effect, thereby reducing the overall strength of the material. The calculation results are consistent with the patterns presented in previous nanoindentation experiments of the A/C structure [52]. The thicker amorphous layer effectively hinders dislocation movement, and the activated dislocation density inside the crystal is low, resulting in softening [52,53,54]. The above analysis quantitatively reveals that the fundamental reason for the decrease in strength of the A/C HEA composite with the increase in amorphous thickness is that the increased amorphous strengthening effect cannot compensate for the reduced crystalline strengthening effect.

## 4. Conclusions

In the work, nanoindentation simulations were conducted on A/C HEA composites using the MD method and a dislocation-based strength model. This study delves into the mechanisms of plastic deformation at the atomic level by analyzing force–penetration depth curves, shear strain, surface topography, structural evolution, and dislocation evolution.

In the elastic deformation, the uneven distribution of elements within the crystal causes heterogeneity in surface tension. The severe mismatch at the interface between amorphous and crystalline materials, coupled with the rapid accumulation of elastic deformation energy, leads to the observation of a large number of plastic deformation behaviors in the traditional sense. The presence of surrounding dislocations restricts the free slip of dislocations below the indenter, while the amorphous layer restricts the possibility of dislocation movement or disappearance towards the substrate. The high-strain region will lead to a composite sound effect on material properties under the coupling effect of the strain gradient and stress gradient. The smaller the thickness of the amorphous layer, the greater the indentation force. At the macroscopic level, it can cause inconsistent stacking and movement trends of surface atoms, resulting in local bulges and depressions. The increase in amorphous layer thickness suppresses the extension of shear bands towards the lower part of the substrate. These results provide a theoretical basis and early guidance for the development of high-performance HEA composite. In the light of the limitations of atomic simulation, there is an urgent need to explore the macroscale deformation mechanisms based on multiscale simulation methods.

## Figures and Tables

**Table 1:** Computational parameters used in the MD simulations.

Materials	Amorphous/Crystalline HEA Composite	Virtual Indenter
Dimensions	464 Å × 455 Å × 460 Å	Radius 100 Å
Number of atoms	5,595,219	
Time step	1 fs	
Initial temperature	300 K	
Indentation velocity	10 m/s	
Depth of indentation	40 Å	
Thickness of amorphous layer 2 nm, 5 nm, 8 nm, and 12 nm		

**Table 2:** Physical parameters of the constituent elements [47,51].

Parameter	Fe	Co	Cr	Ni
Atomic radius (pm)	124	126	125	125
Young’s modulus (GPa)	211	209	279	200
Shear modulus (GPa)	82	75	115	76
Atomic fraction (at%)	25%	25%	25%	25%

**Table 3:** The material parameters of strength models [44,46,47,50].

Parameter	Symbol	Magnitude
Taylor constant	*M*	3
Shear modulus (GPa)	μ	87
Poisson’s ratio	υ	0.3
Burger vector of partial dislocation (nm)	*b_p_*	0.1476
Burger vector of full dislocation (nm)	*b*	0.256
Thickness of amorphous (nm)	t	2, 5, 8, 12
Thickness of crystalline phase (nm)	*d*	44, 41, 38, 34
Average density of dislocation (m^−2^)	ρ	5 × 10^15^~1 × 10^15^
Empirical constant	ξ	0.33
Empirical constant	ψ	1.15

**Table 4:** Values of individual stresses with different amorphous layer thicknesses.

Amorphous Layers Thickness (nm)	$\sigma_{\mathrm{ss}}$ (MPa)	$\sigma_{b}$ (MPa)	$\sigma_{d}$ (MPa)	$\sigma_{A}$ (MPa)
2	270	962	1485	113.5
5	252	949	1206	276
8	233	936	851	439
12	209	917	513	657

## Data Availability

The raw data supporting the conclusions of this article will be made available by the authors on request.
